# Large-scale evacuation route optimization leveraging sampling diversity in quantum annealing

**DOI:** 10.1038/s41598-026-51943-8

**Published:** 2026-05-14

**Authors:** Reo Shikanai, Renichiro Haba, Yusuke Okazaki, Kazumichi Matsumoto, Masayuki Ohzeki

**Affiliations:** 1 Sigma-i Co., Ltd., Tokyo, Japan; 2 Graduate School of Information Sciences, Tohoku University, Sendai, Japan; 3 Sumitomo Corporation, Tokyo, Japan; 4 KYUSHU ELECTRIC POWER CO., INC., Fukuoka, Japan

**Keywords:** Engineering, Mathematics and computing, Natural hazards

## Abstract

In regions such as Japan, where natural disasters frequently occur, it is crucial to evacuate swiftly in the event of a disaster. However, evacuees tend to behave selfishly, that is, they typically head to the nearest shelter along the shortest path. This tendency can lead to severe traffic congestion, thereby exacerbating the damage. To address this issue, we formulate an evacuation route optimization problem that enhances evacuation efficiency as a binary quadratic programming (BQP) problem. The proposed formulation simultaneously minimizes the distance each vehicle must travel to reach a safe location and the penalty associated with overlapping routes among vehicles. In this way, we implicitly aim to reduce the overall completion time of evacuation for all vehicles. For an operational deployment of the proposed method during a disaster, the computation of optimal routes must be completed within a short time; otherwise, it is not practically useful. We therefore investigate the feasibility of employing the quantum annealing machine developed by D-Wave Systems Inc., which has been attracting attention as a promising high-speed solver. Since the current D-Wave machine cannot directly handle large-scale problems that cover an entire city, we design a decomposition method that exploits the intrinsic sampling diversity of the D-Wave machine. We examine the trade-off between solution quality and computation time. Numerical experiments using a traffic simulator demonstrate that the solution of the proposed BQP formulation can shorten the evacuation completion time by up to 33.6% in a specific region of Japan, compared with a locally optimal approach in which all vehicles select the shortest route to the nearest shelter. Although the solution obtained by the proposed decomposition method does not reach the global optimum, it achieves significantly shorter evacuation times than the locally optimal approach, while reducing the computation time drastically. These results are obtained under the assumption that all vehicles strictly follow the computed routes. We further perform simulations under a more realistic assumption in which a fraction of cars choose routes different from those prescribed by the optimization. The results reveal that even if only 1% of vehicles deviate from the optimized routes, the evacuation efficiency deteriorates sharply. Nevertheless, the proposed method still yields a shorter evacuation completion time than the locally optimal approach. These findings suggest that, in time-critical disaster situations, our method provides practical insights for evacuation planning that prioritize rapid and effective action under uncertain conditions, rather than insisting on strict optimality.

## Introduction

Japan is frequently struck by natural disasters, including earthquakes, tsunamis, and volcanic eruptions. In July 2017, for example, heavy rainfall struck the Kyushu region, resulting in 37 fatalities and four missing persons. A review article on evacuation planning^1^ points out that, in such disaster situations, evacuees tend to behave selfishly: they move along the shortest paths to the nearest shelters. In the Great East Japan Earthquake of 2011, many residents evacuated by car, which is believed to have caused traffic congestion and possibly amplified the damage^2^. Municipalities in Miyagi Prefecture currently provide smartphone applications that indicate the nearest shelter from the user’s present location. However, evacuees are still required to make flexible decisions as to which shelter to head for and by which route. Even if the locations of evacuees could be obtained immediately after a disaster, computing optimal evacuation routes in real time would be computationally expensive. Moreover, during evacuation, road conditions can change rapidly due to river flooding, fallen trees, road closures, and congestion, making it necessary to repeatedly perform route optimization within a short time.

One promising option for such rapid combinatorial optimization is quantum annealing (QA). The quantum annealing machine developed by D-Wave Systems Inc. has attracted considerable attention as the world’s first commercial quantum computing device. It is designed to solve combinatorial optimization problems by a technique known as quantum annealing (QA). While simulated annealing (SA) uses thermal fluctuations, with uphill moves accepted with probabilities that are exponentially suppressed as $\exp (-\Delta E/T)$, QA explores the solution space by introducing quantum fluctuations through a transverse field^3^. This transverse field can induce tunneling between configurations, which is often argued to be beneficial when the energy landscape contains tall, narrow barriers that are difficult to overcome by purely thermal activation^3,4^. Accordingly, QA may offer an advantage over SA on problem classes exhibiting local ruggedness, although such an advantage is problem-dependent rather than universal^3,4^. These properties make QA attractive for evacuation route optimization, where many interacting route choices must be coordinated quickly. QA has also been applied to traffic-related optimization and to hybrid methods that combine quantum and classical computation^5–10^. Nevertheless, directly applying QA to a city-scale evacuation problem remains difficult in practice.

The latest quantum annealing machine from D-Wave, named ”Advantage2,” provides approximately 5,000 qubits. To solve a combinatorial optimization problem on a D-Wave machine, however, one must perform a preprocessing step known as minor embedding, which maps the logical graph of the problem onto the hardware graph formed by the physical qubits. During embedding, a single logical variable is represented by multiple physical qubits, which are chained together to reproduce the connectivity among logical variables on the hardware. When the logical connectivity cannot be directly realized on the hardware graph, the number of physical qubits included in each chain increases, and the chains become longer. As chains grow, more physical qubits are consumed merely to maintain logical variables, and thus the adequate size of problems that can be handled becomes significantly smaller than the total number of qubits installed on the device.

To address this hardware limitation and tackle larger-scale problems, decomposition-based hybrid approaches that combine classical computers with quantum annealers have been proposed. Early examples include frameworks such as qbsolv^11^, which apply quantum annealing to subproblems extracted from a large quadratic unconstrained binary optimization (QUBO) problem and iteratively update a global solution using classical optimization techniques. More recently, general-purpose hybrid solvers developed by D-Wave Systems have become available for solving large constrained binary quadratic problems. In practice, however, when dealing with city-scale evacuation routing problems involving thousands to tens of thousands of vehicles, even such hybrid solvers can require substantial computation time to solve a single instance. This is a serious limitation in disaster response, where solutions must be obtained rapidly and may need to be recomputed as road conditions and traffic states change. We therefore focus on the ability of quantum annealing machine to rapidly obtain multiple diverse low-energy solutions, and consider that this property can be exploited within a decomposition-based hybrid approach.

An important characteristic of quantum annealing is its intrinsic sampling capability. Unlike classical deterministic solvers that output a single solution, a quantum annealer functions as a sampler that probabilistically generates multiple solutions in each run. By performing multiple anneals, one can obtain not only the optimal solution but also a variety of high-quality near-optimal solutions. Zucca et al. demonstrated that the D-Wave machine can sample diverse low-energy solutions more rapidly than classical solvers^12^. In this study, we propose a general decomposition framework that exploits this sampling capability. Specifically, multiple low-energy candidate solutions are generated for each sub-QUBO, and their selection and combination are formulated as an auxiliary optimization problem. This design keeps the computation practical for large-scale evacuation problems while still using information from multiple low-energy samples. To evaluate the intrinsic effectiveness of the proposed BQP formulation itself, the original, non-decomposed problem is also solved using a D-Wave hybrid solver. This non-decomposed solution serves as a benchmark that reflects the performance of the formulation without problem-specific decomposition, allowing a clear assessment of the trade-off between solution quality and computation time introduced by the proposed decomposition strategy.

Based on this idea, we formulate evacuation route optimization as a BQP and evaluate the formulation using both a D-Wave machine and a commercial classical solver, comparing their performance in terms of solution quality and computation time. The key methodological contribution is not merely to decompose the original problem, but to reformulate it as a selection problem over sampled candidate solutions of clustered sub-QUBOs. More specifically, the original route-assignment BQP is converted into an auxiliary quadratic optimization problem that selects one candidate solution from each cluster while explicitly retaining cross-cluster route-overlap interactions. We then investigate the trade-off between computation time and solution quality when this decomposition method is applied to large-scale instances. The objective of the proposed approach is not to achieve strict optimality, but to obtain practically reasonable solutions rapidly under the severe time constraints inherent in disaster response. In addition, simulations incorporating realistic uncertainty, in which some vehicles do not follow the optimized routes, are conducted to examine whether the improvement in evacuation efficiency over conventional approaches can be maintained even when the system partially fails to operate as intended.

The remainder of this paper is organized as follows. In the next section, building on a previous study on congestion mitigation for taxi traffic^5^, we propose a formulation of the evacuation route optimization problem under disaster conditions. We also introduce a decomposition method that enables the use of a D-Wave machine for large-scale problems by exploiting its sampling diversity. We evaluate the proposed method primarily by comparing evacuation completion times for all vehicles. Furthermore, we conduct experiments under a realistic simulation scenario in which some vehicles do not follow the optimized routes. Finally, we conclude the paper.

## Methods

In this section, we describe the formulation of the evacuation route optimization problem under a disaster scenario. The formulation is generic and not limited to a specific type of disaster. For concreteness, we consider flooding as an illustrative example. In the case of flooding, vehicles located inside a hazard area must evacuate as quickly as possible either to outside the hazard area or to safe facilities within the area. To achieve this, evacuation routes must be diversified so as to avoid traffic congestion. Neukart et al. proposed a quadratic unconstrained binary optimization (QUBO) formulation whose purpose is to diversify routes and thereby alleviate congestion in taxi traffic^5^. We build upon that QUBO and introduce several modifications so that it can be applied to the evacuation route optimization.

We represent the city map as a graph, where intersections are nodes and road segments are edges. Routes are thus represented as paths on this graph. In the QUBO proposed in the previous work^5^, the objective is to minimize the number of overlapping edges among candidate routes. In the present evacuation route optimization problem, however, we must not only diversify the routes but also ensure that vehicles reach safe areas as quickly as possible. For example, if a vehicle is located near the boundary of a hazard area, an efficient evacuation strategy is to first leave the hazard area as rapidly as possible and then proceed safely and at a lower speed toward a shelter outside the area. With this in mind, we formulate the problem as follows. Because the purpose of this study is to provide each vehicle with a specific evacuation route, we adopt a vehicle-level formulation in which the decision variable indicates which candidate route is selected for each vehicle.

1 $$\begin{aligned} \begin{aligned} \min _{\vec {x}} F(\vec {x})=&\;\alpha \sum _{i \in V} \sum _{j \in R_i} d_{ij} x_{ij}+ \sum _{\begin{array}{c} i, k \in V \\ i \ne k \end{array}} \sum _{j \in R_i} \sum _{l \in R_k} c_{ijkl} x_{ij} x_{kl}, \\ \text{ s.t. }&\quad \sum _{j \in R_i} x_{ij}=1, \quad \forall i \in V, \\&\quad x_{ij} \in \{ 0, 1\}, \quad \forall i \in V, \; \forall j \in R_i \;, \end{aligned} \end{aligned}$$

where $x_{ij}=1$ is a binary variable indicating that vehicle *i* selects candidate route *j*. The set *V* denotes the indices of vehicles subject to evacuation, and $R_i$ is the index set of candidate routes for vehicle *i*. Thus, candidate routes must be generated in advance; the specific procedure will be described later. The quantity $d_{ij}$ represents the distance that vehicle *i* must travel along candidate route *j* until it reaches a safe state. For instance, if the destination shelter lies inside the hazard area, $d_{ij}$ is the route distance from the current location of the vehicle to the shelter. If, on the other hand, the shelter lies outside the hazard area, $d_{ij}$ is defined as the route distance until the vehicle exits the hazard area. In this study, we assume that the risk to human life is largely mitigated once a vehicle exits the hazard area, and therefore, we do not explicitly consider travel distance or congestion risk outside the hazard area in the cost function. Minimizing the first term thus encourages each vehicle to reach a safe area by the shortest path. The quantity $c_{ijkl}$ denotes the number of overlapping road segments between candidate route *j* of vehicle *i* and candidate route *l* of vehicle *k*. Minimizing the second term serves to diversify routes and thereby prevent congestion. By minimizing this objective function $F(\vec {x})$, we aim to indirectly improve the overall evacuation completion time until all vehicles reach safe areas, by simultaneously controlling travel distances and avoiding excessive concentration on the same road segments. Here, the evacuation completion time itself is not directly included in the optimization model, because it depends on dynamic traffic interactions and delays caused by traffic signals and can be evaluated only after running a traffic simulation. In addition, in a real disaster, it is generally difficult to obtain or accurately predict the true overall evacuation completion time at the moment when route guidance must be issued. Instead, the proposed objective function is used as a surrogate: the first term favors routes that lead vehicles to safety quickly, and the second term discourages excessive overlap among routes that may cause congestion. Nevertheless, this modeling choice introduces a limitation of the formulation itself, because minimizing $F(\vec {x})$ does not guarantee minimization of the true evacuation completion time. The hyperparameter $\alpha$ is a real-valued weight that balances these two objectives. Larger values of $\alpha$ place more emphasis on the travel-distance term, whereas smaller values of $\alpha$ relatively emphasize the route-overlap penalty.

2 $$\begin{aligned} \begin{aligned} \min _{\vec {x}} G(\vec {x})=&\; \alpha \sum _{i \in V} \sum _{j \in R_i} d_{ij} x_{ij}+ \sum _{\begin{array}{c} i, k \in V \\ i \ne k \end{array}} \sum _{j \in R_i} \sum _{l \in R_k} c_{ijkl} x_{ij} x_{kl}+\beta \sum _{i}\left( \sum _{j \in R_i}x_{ij} - 1 \right) ^2, \\&\text{ s.t. } \quad x_{ij} \in \{ 0, 1\}, \quad \forall i \in V, \; \forall j \in R_i \;, \end{aligned} \end{aligned}$$

where $\beta$ is a real-valued hyperparameter that serves as a penalty coefficient for the constraint term. The penalty reformulation in Eq. (2) does not inherently undermine optimality: if $\beta$ is sufficiently large and Eq. (2) is solved exactly, the optimal solution of Eq. (2) coincides with the optimal feasible solution of the original constrained problem in Eq. (1). By contrast, if $\beta$ is too small, infeasible assignments may attain a lower penalized objective value, and the equivalence to Eq. (1) can be lost. In practice, Eq. (2) may also be solved only approximately because of solver limitations and time constraints. In the present study, we fix $\beta =10$ and tune only $\alpha$, so that the influence of the trade-off between travel-distance reduction and route dispersion can be examined under a penalty coefficient chosen to preserve feasibility in the instances considered here.

When solving Eq. (2) on a D-Wave machine, minor embedding becomes increasingly complex as the problem size grows. Because evacuation route optimization must cover a large area, such as an entire disaster-stricken city, the original QUBO will often be too large to be directly embedded on the hardware. To address this issue, several methods have been proposed in which a QUBO is decomposed into smaller subproblems. For example, in some decomposition-based approaches, such as qbsolv^11^, a large QUBO is split into sub-QUBOs whose sizes are small enough to be embedded on a D-Wave machine, and variables that are not included in each sub-QUBO are clamped. The extracted sub-QUBOs are then solved using a D-Wave machine or other solvers. In a study on traffic signal optimization^6^, the graph partitioning tool Metis^13^ was used to group strongly interacting logical variables into the same sub-QUBO, and the minimum-energy solution of each sub-QUBO was computed.

In these methods, the solution of the original QUBO is constructed by taking only the minimum-energy solution from each sub-QUBO and combining them. However, there is no guarantee that the minimum-energy solution of a sub-QUBO always forms a part of the global optimum of the original QUBO. When interactions between sub-QUBOs are strong, selecting only the minimum-energy solutions of the sub-QUBOs may result in a combination that is suboptimal for the original problem. To mitigate this limitation, we propose a decomposition method in which multiple solutions are sampled from each sub-QUBO, and the collection of these solutions is then used as input to a separate optimization problem that determines the global solution.

The novelty of the proposed decomposition method lies in replacing the original variable-level optimization with a candidate-solution-level optimization over clustered sub-QUBOs. That is, instead of directly optimizing all route-assignment variables in the original BQP, we first generate multiple candidate solutions for each cluster and then solve an auxiliary quadratic problem that selects and combines them. This reformulation explicitly preserves cross-cluster interaction costs between candidate solutions, and therefore differs from a simple decomposition approach in which each sub-QUBO is solved independently and only their locally best solutions are combined.

The proposed framework differs from column generation in that the candidate solutions are not iteratively generated and updated during the optimization process. Instead, multiple candidates are sampled in advance for each sub-QUBO and are then combined through an auxiliary optimization problem. This avoids repeated re-optimization and is therefore advantageous in situations where rapid computation is required.

Before detailing the decomposition procedure, we briefly describe the mathematical and physical basis by which the D-Wave quantum processing unit (QPU) generates multiple candidate solutions. In the closed-system approximation, the programmed QA dynamics is described by the time-dependent Hamiltonian

3 $$\begin{aligned} \hat{H}(s) = -A(s)\sum _i \hat{\sigma }_i^x + B(s)\hat{H}_P , \end{aligned}$$

where $s=t/t_a\in [0,1]$ is the normalized annealing time, *A*(*s*) and *B*(*s*) are the annealing schedules, $\hat{\sigma }_i^x$ is the Pauli-*X* operator acting on qubit *i*, and $\hat{H}_P$ is the Ising problem Hamiltonian obtained from the QUBO in Eq. (2) via the standard binary-to-spin mapping. In the ideal adiabatic limit, the system remains near the instantaneous ground state and the final state encodes a minimum-energy solution of $\hat{H}_P$.

In practice, however, the QPU operates as an open quantum system at finite temperature, with finite annealing time and control errors. Therefore, non-adiabatic transitions and thermal effects can lead to a distribution of measured bitstrings rather than a single deterministic ground-state output. Phenomenologically, this output distribution is often approximated by an effective Gibbs-like form,

4 $$\begin{aligned} P(\textbf{s}) \approx \frac{1}{Z}\exp \!\left[ -\beta _{\textrm{eff}} H_P(\textbf{s})\right] , \end{aligned}$$

where $\textbf{s}\in \{\pm 1\}^n$ denotes a spin configuration, $\beta _{\textrm{eff}}$ is an effective inverse temperature, and *Z* is the partition function. Repeated anneals therefore generate a pool of low-energy samples, including the ground state and low-lying excited states. Following Zucca et al., the diversity of such samples can be understood in terms of their mutual separation in Hamming space among high-quality solutions. In this study, we exploit this distributional sampling property to obtain multiple distinct candidate route assignments for each sub-QUBO, from which a retained subset is constructed for the subsequent merging optimization.

First, we partition the set of vehicles into *M* clusters and denote the set of cluster indices by $C=\{1,2,\dots ,M\}$.

Let $V^{m} = \{i_{1}^{m}, i_{2}^{m}, \dots , i_{N^{m}}^{m}\}$ be the index set of vehicles belonging to cluster *m*. For each cluster, we construct a sub-QUBO based on Eq. (2). Next, we assign a unique label to every feasible solution of the sub-QUBO for cluster *m* and denote the set of all such labels by $X^{m}$. In other words, $X^{m}$ represents the index set of all candidate solutions of the sub-QUBO for cluster *m*. Here, we assume that each feasible solution in $X^{m}$ satisfies the one-hot constraints $\sum _{j \in R_i} x_{ij}^{mn} = 1$ for all $i \in V^{m}$ (with $x_{ij}^{mn} \in \{0,1\}$). We then introduce a binary variable $s_{mn}$ that takes the value 1 if and only if the *n*-th feasible solution in $X^{m}$ is selected. This reformulation does not introduce an approximation to Eq. (2); rather, it changes the representation of the decision variables from route-level to solution-selection binary variables. Since each sub-QUBO admits only a finite number of feasible solutions, the original route-level binary variables $\vec {x}$ are implicitly represented by the selection variables $\{s_{mn}\}$. Under this representation, Eq. (1) can be equivalently rewritten as a minimization problem over $\vec {s}$, as shown in Eq. (5).

5 $$\begin{aligned} \begin{aligned} \min _{\vec {s}} \; H(\vec {s}) =&\sum _{m \in C} \sum _{n \in X^{m}} \left( \alpha \sum _{i \in V^{m}} \sum _{j \in R_i} d_{ij} x_{ij}^{mn} + \sum _{\begin{array}{c} i,k \in V^{m} \\ i \ne k \end{array}} \sum _{j \in R_i} \sum _{l \in R_k} c_{ijkl} x_{ij}^{mn} x_{kl}^{mn} \right) s_{mn} \\&\quad + \sum _{\begin{array}{c} m,p \in C \\ m \ne p \end{array}} \sum _{n \in X^{m}} \sum _{q \in X^{p}} \sum _{i \in V^{m}} \sum _{j \in R_i} \sum _{k \in V^{p}} \sum _{l \in R_k} c_{ijkl} x_{ij}^{mn} x_{kl}^{pq} s_{mn} s_{pq}, \\ \text {s.t.} \quad&\sum _{n \in X^{m}} s_{mn} = 1, \quad \forall m \in C, \\&s_{mn} \in \{0,1\}, \quad \forall m \in C,\; \forall n \in X^{m}. \end{aligned} \end{aligned}$$

Here, the first term represents the linear coefficients corresponding to the cost within each sub-QUBO (distance to safety and route-overlap penalties among vehicles in the same cluster). The second term represents the quadratic interaction between vehicles belonging to different clusters, where the coefficients are defined based on the number of overlapping road segments between their routes. Intuitively, this formulation can be interpreted as follows: for each cluster *m*, we select exactly one solution from the candidate solutions prepared in advance, and determine the binary variables $s_{mn}$ so that the resulting combination of selected solutions minimizes congestion at the global level. At this stage, the detailed route choices within each cluster are encapsulated in $\vec {x}^{mn}$, and $H(\vec {s})$ functions as a QUBO that optimizes the combination of candidate solutions selected in each cluster. This formulation is equivalent to the original constrained formulation in Eq. (1) when all feasible solutions are included in $X^{m}$, and becomes an approximation when only a sampled subset $\bar{X}^{m} \subseteq X^{m}$ is used. In practice, however, enumerating all feasible solutions in $X^{m}$ is computationally intractable for large-scale instances.

We refer to the vector $\vec {x} \in \{0,1\}^{N}$ corresponding to the minimum of $H(\vec {s})$ as the “merged solution.” The merged solution is constructed by combining, for each cluster *m*, the partial solution $\vec {x}^{m n^{*}}$ selected in the minimum-energy solution $\vec {s}$ of $H(\vec {s})$, as

6 $$\begin{aligned} \vec {x} = \sum _{m=1}^{M} S^{m} \, \vec {x}^{m n^{*}} . \end{aligned}$$

Here, $S^{m}$ is a reconstruction matrix that maps the partial solution $\vec {x}^{m n^{*}}$ for cluster *m* back to the corresponding indices of the original solution vector $\vec {x}$. If we define the index set of variables belonging to cluster *m* as $I_m = \{i_1, i_2, \dots , i_{N_m}\}$, then the reconstruction matrix can be written using the standard basis vectors $\vec {e_i}$ as $S^{m} = [\, \vec {e_i} \mid i \in I_m \,]$, where $\vec {e_i}$ is the standard basis vector whose *i*-th component is 1 and all others are 0.

For example, suppose that the full solution vector is

$$\vec {x} = (x_1, x_2, x_3, x_4)^{T}.$$

Suppose that it is partitioned into

$$\vec {x}^{1} = (x_1, x_4)^{T}, \quad \vec {x}^{2} = (x_2, x_3)^{T}.$$

The reconstruction matrices are then given by

$$S^{1} = \left( \begin{array}{ll} 1 & 0 \\ 0 & 0 \\ 0 & 0 \\ 0 & 1 \end{array}\right) , \quad S^{2} = \left( \begin{array}{ll} 0 & 0 \\ 1 & 0 \\ 0 & 1 \\ 0 & 0 \end{array}\right) .$$

In general, the total number of feasible solutions for the sub-QUBO of cluster *m* is $|X^{m}| = \prod _{i \in V^{m}} |R_i|$, because each vehicle *i* selects exactly one route from its candidate set $R_i$, and thus the product of the numbers of candidate routes for all vehicles in the cluster coincides with the total number of candidate solutions for the sub-QUBO.

In the quadratic term of Eq. (5), we must define interaction coefficients for all solution pairs $(n,q) \in X^{m} \times X^{p}$ between clusters *m* and *p*. Thus, the total number of required quadratic coefficients is of order $\sum _{m \ne p} |X^{m}|\,|X^{p}|$, and since each $|X^{m}|$ itself grows drastically with the number of vehicles, the precomputation of these coefficients becomes prohibitively expensive when many vehicles are present. Moreover, it is impractical in general to enumerate all feasible solutions of each sub-QUBO.

In practice, therefore, we use only a representative subset $\bar{X}^m \subseteq X^m$ of candidate solutions for each cluster *m*, obtained by sampling, where $|\bar{X}^m| \le |X^m|$. Here, the retained candidates are restricted to feasible solutions satisfying the one-hot constraints. That is, for each cluster, we sample $|\bar{X}^m|$ candidate solutions, and compute the linear and quadratic coefficients only for the solutions contained in these subsets, thereby significantly reducing the computational cost of coefficient calculation. On the other hand, restricting candidate solutions to $\bar{X}^m$ implies that, if the partial solutions that constitute the global optimum of the original QUBO are not included in $\bar{X}^m$, then the true global optimum cannot be reached, regardless of how accurately Eq. (5) is solved. Consequently, the choice of $|\bar{X}^m|$ involves a trade-off between computation time (for coefficient calculation and optimization) and the quality of the final solution. In this study, we prioritize the reduction of computation time within this trade-off and adopt a construction that uses relatively small subsets $\bar{X}^m$. Nevertheless, as shown in the numerical experiments presented later, the proposed method yields better solutions than a locally optimal baseline, despite this approximation.

To summarize, the proposed decomposition method consists of the following steps.

1. Cluster vehicles into *M* clusters $\{V^{m}\}_{m=1}^{M}$ by applying *K*-means clustering to their two-dimensional coordinates.
2. For each cluster *m*, construct a sub-QUBO based on Eq. (2), sample candidate solutions using a sampler such as quantum annealing, and extract the unique low-cost feasible solutions satisfying the one-hot constraints as the candidate set $\bar{X}^{m}$.
3. Using the sampled solution sets $\{\bar{X}^{m}\}_{m=1}^{M}$, solve the minimization problem in Eq. (5) and reconstruct the final solution vector $\vec {x}$ via Eq. (6).

The proposed decomposition method aims to cluster vehicles with strong mutual interactions into the same cluster. By doing so, major interaction terms representing route overlaps can be locally optimized within each sub-QUBO, while the contribution of interaction terms spanning different clusters can be suppressed during the merging stage. To implement this, we adopt clustering based on the two-dimensional coordinates of vehicles as a computationally efficient heuristic. This choice is motivated by the process used to generate candidate routes. Since each vehicle’s candidate routes are computed with nearby shelters as destinations, vehicles that are geographically close to one another are likely to share similar candidate routes and head toward the same group of shelters. In other words, vehicles that are spatially close tend to traverse the same road segments and thus interact strongly. While interaction-based clustering would be ideal, it would require evaluating pairwise interactions between all vehicles, leading to a significantly higher computational cost. Therefore, we use coordinate-based clustering as a lightweight approximation that tends to confine major interaction terms within each sub-QUBO under strict time constraints. The following section evaluates both the improvement in evacuation efficiency achieved by the proposed BQP/QUBO formulation and the reduction in computation time realized by the decomposition method.

## Results

In this section, we evaluate whether the solutions to Eqs. (1) and (2) can improve evacuation efficiency. For this purpose, we employ Simulation of Urban MObility (SUMO)^14^, an open-source traffic simulator. In SUMO, vehicles move autonomously on a road network derived from map data such as OpenStreetMap. When a leading vehicle stops, following vehicles form a queue and traffic congestion emerges naturally. The simulator also supports traffic lights, and vehicle movements are controlled according to the signal states. Since map data from OpenStreetMap and related sources may be incomplete, SUMO provides interpolation capabilities. For example, if the cycle times of traffic lights are not specified, SUMO automatically infers them based on road length and other information.

We consider flood disasters and conduct simulations on two geographical models in Asakura City, Fukuoka Prefecture, Japan: a small-area model and a large-area model. Asakura City suffered severe damage due to heavy rainfall in July 2017. In both models, vehicle locations are generated based on real-world household distribution data derived from residential statistics published by the national government via the Portal Site of Official Statistics of Japan (e-Stat). In the small-area model, only part of Asakura City is designated as the evacuation target. For this small-scale setting, evacuation is assumed to be conducted on a per-household basis, and each household is represented by a single vehicle. Under this assumption, the total number of vehicles is limited to approximately 200 in order to enable the use of the Direct QPU of the D-Wave system within a decomposition-based solution framework. In the large-area model, the entirety of Asakura City and the neighboring town of Tachiarai are considered as the evacuation target. In this setting, vehicle locations are also generated based on household distribution data, resulting in approximately 16,600 vehicles. A major river flows between Asakura City and Tachiarai Town, and flooding of this river during heavy rainfall events has caused severe damage, which is why this region is selected for the experiments. Shelter locations are based on publicly available evacuation shelter information provided by the municipal governments of Asakura City and Tachiarai Town. Hazard areas are derived from real-world hazard data obtained via e-Stat. Base map data are obtained from OpenStreetMap.

For each vehicle, we generate four candidate routes using Dijkstra’s algorithm. This number was chosen as a minimal setting that can represent both destination choice and route dispersion. A larger number of candidate routes would substantially increase the size of the optimization problem. Specifically, we first select, in order of proximity, two shelters as candidate destinations for each vehicle and compute the shortest paths from the vehicle’s current position to each shelter using Dijkstra’s algorithm, adding them to the set of candidate routes. Next, we increase the weights of the edges included in the computed shortest paths and rerun Dijkstra’s algorithm to obtain alternative detour routes to each shelter. Specifically, the weights of these edges are multiplied by a factor of 1.1. This factor is chosen so that the resulting routes deviate slightly from the shortest paths while avoiding excessively long detours that would never be selected as reasonable evacuation routes. Thus, each vehicle is assigned two candidate shelters and, for each shelter, two candidate routes consisting of a shortest route and a detour route. In this way, each vehicle is provided with four candidate routes in total.

In addition to the two geographical models, we consider two types of evacuation scenarios: an idealized scenario and a realistic scenario. The idealized scenario assumes that all vehicles follow the routes given by the optimization without deviation; this setting is used to evaluate the intrinsic efficiency of the proposed BQP-based routing. The realistic scenario, in contrast, allows for the possibility that vehicles do not necessarily follow the optimized routes. In actual disaster situations, even if optimal routes are presented to residents, it is natural to assume that some evacuees will behave selfishly–choosing the shortest route to the nearest shelter–or otherwise fail to follow the recommended routes for various reasons. We investigate how the presence of such vehicles, at a certain fraction of the total, affects evacuation efficiency. As a metric of evacuation efficiency, we measure the time until all vehicles complete evacuation, which we refer to as the *evacuation completion time*. Vehicles aim at designated shelters, but when a shelter is located outside the hazard area, we count the evacuation as complete when a vehicle exits the hazard area. If a shelter is inside the hazard area, we regard evacuation as complete when the vehicle arrives at that shelter.

**Fig. 1:**
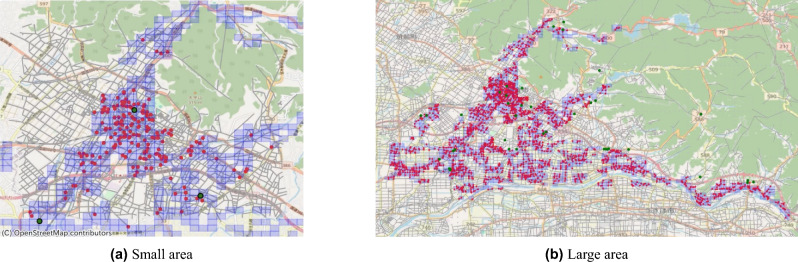
Maps used in the experiments. (**a**) Small-area model (a subregion of Asakura City, Fukuoka Prefecture) and (**b**) large-area model (entire Asakura City and Tachiarai Town, Fukuoka Prefecture). Red dots represent vehicles generated based on the spatial distribution of households, green dots denote shelters, and the blue mesh indicates the hazard area. The plots illustrate the initial state of the simulations. The maps were created by the authors using Python 3.13.0 (https://www.python.org/), OSMnx 1.9.3 (https://osmnx.readthedocs.io/), Contextily 1.6.0 (https://contextily.readthedocs.io/), and Matplotlib 3.9.0 (https://matplotlib.org/). Road network data were obtained from OpenStreetMap using OSMnx. The base map tiles were added using Contextily with the OpenStreetMap Mapnik provider. Base map data © OpenStreetMap contributors, available under the Open Database License at https://www.openstreetmap.org/copyright. Hazard data and household distribution data were extracted from the Portal Site of Official Statistics of Japan (e-Stat), jSTAT MAP. Shelter locations were obtained from publicly available evacuation shelter information provided by the municipal governments of Asakura City and Tachiarai Town. All datasets were processed and plotted by the authors.

For solving the optimization models, we use the branch-and-bound-based Gurobi Optimizer^15^, simulated annealing (SA) implemented in OpenJij^16^, and the quantum annealing machines developed by D-Wave Systems Inc. In particular, for the small-area model, we employ the Advantage2 system, which uses only physical qubits, whereas for the large-area model, we use the D-Wave Hybrid Constrained Quadratic Model (CQM) solver, which performs hybrid computation combining classical processors with quantum annealing. The hybrid CQM solver can handle constrained binary quadratic problems with up to 500,000 variables (Fig. 1).

The hyperparameters in the QUBO were chosen by grid search. We fixed $\beta = 10$ so that the one-hot constraints were satisfied with high probability, and tuned only $\alpha$ over the range $\alpha \in \{1,3,5,\ldots ,19\}$. Among these values, $\alpha = 7$ yielded the smallest evacuation completion time. This result suggests that, for the road networks used in this study, placing a relatively large weight on the travel-distance term while still penalizing route overlap leads to better evacuation efficiency. At the same time, the best value of $\alpha$ may depend on the structure of the map. All classical computations with Gurobi and OpenJij SA were performed on a machine equipped with two Intel Xeon Gold 6338 CPUs and 256 GB of memory.

**Fig. 2:**
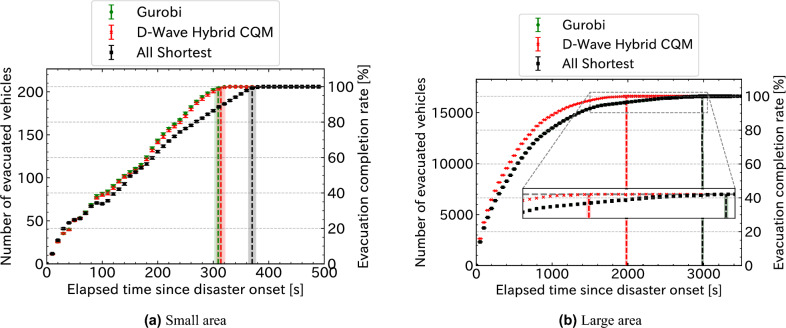
Comparison of evacuation completion times for the small and large areas. (**a**) Progress of evacuation in the small-area model (approximately 200 vehicles) and (**b**) in the large-area model (approximately 16,600 vehicles). Each plot shows, on the left axis, the number of evacuated vehicles and, on the right axis, the evacuation completion rate. Markers represent averages over multiple simulation instances, and the associated error bars indicate the standard error. The proposed method solved by Gurobi and by the D-Wave Hybrid CQM solver is compared with the baseline in which all vehicles move along the shortest route to the nearest shelter (All Shortest). Vertical dashed lines indicate the mean evacuation completion time for each method, and the shaded bands around them represent the corresponding standard error. In the large-area model, the D-Wave Hybrid CQM solver reduces the evacuation completion time by about 33.6% compared with the baseline.

We first examine whether the proposed formulation can, in fact, improve evacuation efficiency. Under the idealized scenario where all vehicles strictly follow the optimized routes, Fig. 2 shows the results of solving Eq. (1) without decomposition. Figure 2a and 2b correspond to the small-area and large-area models, respectively. We use Gurobi Optimizer and the D-Wave Hybrid CQM solver as optimization engines. As a baseline, we adopt a behavior pattern that is plausible during disasters, namely, that all vehicles head to the nearest shelter via the shortest path; we refer to this baseline as ”All Shortest.” For the small-area model, each data point represents the mean over 10 random instances, and the error bar shows the standard error. The time limit for each solver was set to 5s. The results show that Gurobi and the hybrid CQM solver achieve almost the same evacuation completion time, and the slightly better Gurobi solution reduces the evacuation completion time by approximately 16.5% compared with All Shortest. For the large-area model, we report the mean and standard error over six instances, with the time limit for each solver set to 400 s. In this case, Gurobi yields almost the same result as All Shortest. This is because Gurobi, as a branch-and-bound-based solver, starts from the initial solution where all vehicles move along the shortest path to the nearest shelter and fails to find a better solution within the time limit. In contrast, the D-Wave Hybrid CQM solver achieves an improvement of approximately 33.6% compared with All Shortest. These results confirm that the proposed BQP formulation can enhance evacuation efficiency. The larger improvement in the large-area model is presumably due to the higher vehicle density in the road network, which amplifies the effect of the route diversification term in the cost function.

**Fig. 3:**
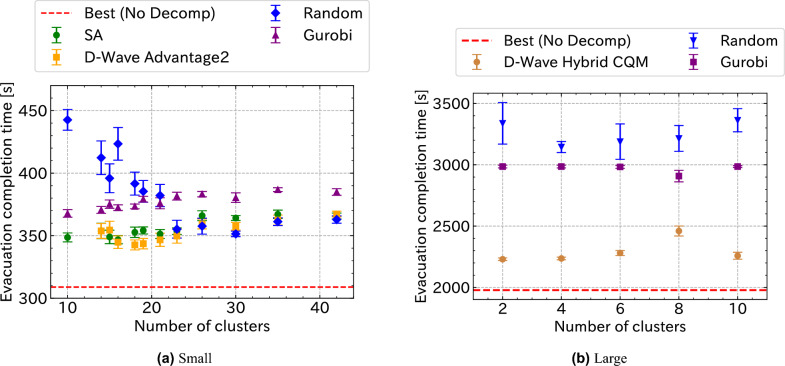
Evacuation completion time as a function of the number of clusters. (**a**) Small-area model (approximately 200 vehicles) and (**b**) large-area model (approximately 16,600 vehicles). Markers represent averages over multiple simulation instances, and the associated error bars indicate the standard error. We compare the case where solutions are sampled uniformly at random from each cluster (Random) with cases where low-cost solutions are sampled using SA, D-Wave Advantage2/Hybrid CQM, or Gurobi. The horizontal dashed line labeled Best (No Decomp) indicates the instance-averaged evacuation completion time obtained by solving the original problem without decomposition. In both models, solver-based sampling yields better evacuation completion times than random sampling, but none of the decomposition-based methods reach the instance-averaged best solution obtained without decomposition.

We next investigate the performance of the proposed decomposition method. The results are shown in Fig. 3. Again, we consider the idealized scenario. Panel a corresponds to the small-area model, and panel b to the large-area model. In Fig. 3a, we use Gurobi Optimizer, D-Wave Advantage2, the Hybrid CQM solver, and OpenJij SA. For D-Wave Advantage2 and OpenJij SA, we sample 1,000 solutions for each cluster *m* and retain the 10 unique lowest-cost feasible solutions as $\bar{X}^m$. In addition, we generate 1,000 uniformly random solutions for each cluster and select the 10 solutions with the lowest cost; we refer to this sampling scheme as ”Random.” In contrast, for Gurobi-based sampling, we do not perform stochastic sampling; instead, for each cluster *m*, we use the (near-)optimal solution returned by Gurobi as the single candidate solution in $\bar{X}^m$. Figure 3a plots the mean and standard error over 10 instances, whereas Fig. 3b does so over five instances. The horizontal axis indicates the number of clusters, and the vertical axis the evacuation completion time. In both the small-area and large-area models, none of the solvers achieves a shorter evacuation completion time than that obtained without decomposition.

**Fig. 4:**
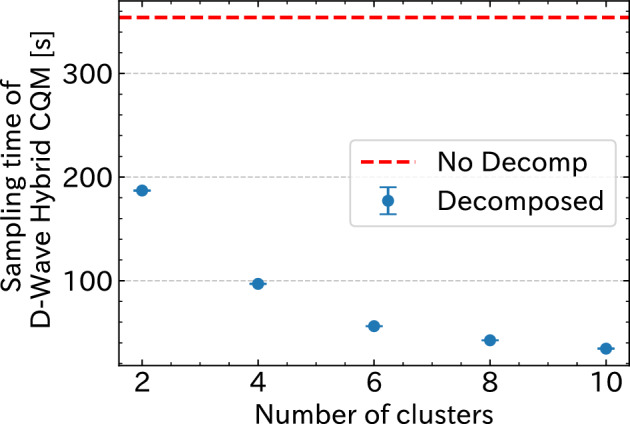
Minimum computation time of the D-Wave Hybrid solver in the decomposition method. The D-Wave Hybrid CQM solver automatically determines the minimum required computation time based on the size of the input problem. Markers represent averages over multiple instances of the minimum computation time assigned by the solver, and the associated error bars indicate the standard error. This figure shows the instance-averaged minimum computation time as a function of the number of clusters. As the number of clusters increases, each sub-QUBO becomes smaller, and the required minimum computation time decreases significantly. The horizontal dashed line labeled No Decomp indicates the instance-averaged minimum computation time when no decomposition is applied. In this case, the problem size becomes large, leading to a substantially longer minimum computation time, which is impractical for time-critical evacuation scenarios.

In the large-area model shown in Fig. 3 b, we compare CQM-based sampling with Gurobi-based sampling and random sampling. For the CQM solver, the minimum time limit is automatically determined by the problem size at run time; we use this minimum time as the sampling time. When no decomposition is applied, the Hybrid CQM solver requires a minimum computation time of approximately five minutes for the large-area model. While CQM-based sampling with decomposition yields shorter evacuation completion times than Gurobi-based or random sampling, it still does not reach the performance of the solution obtained without decomposition. By contrast, solving the problem without decomposition using the Hybrid CQM solver entails a substantially longer computation time, which is impractical for time-critical evacuation planning. We discuss the reasons for the performance gap in the next section. We also observe that the evacuation completion time hardly changes with the number of clusters. Figure 4 shows the sampling time of the CQM solver as a function of the number of clusters. The plotted values correspond to the case where sampling for each cluster is performed in parallel. As the number of clusters increases, the size of each sub-QUBO decreases, and the required minimum computation time decreases drastically. On the other hand, since the evacuation completion time is almost independent of the number of clusters, there are cases in which the decomposition method becomes more favorable than solving the original problem without decomposition, from the viewpoint of the trade-off between computation time and solution quality. In particular, for evacuation route optimization where time is critically limited, computational speed is often more important than marginal improvements in solution quality.

So far, we have presented simulation results under the idealized scenario. We now describe the experimental setup for the realistic scenario. In real evacuation behavior, even if optimized routes are presented to evacuees, it is reasonable to assume that a certain fraction of vehicles behaves selfishly and instead follows the shortest path to the nearest shelter. In our experiment, we evaluate the robustness of the solutions by investigating how the evacuation completion time changes when such selfish vehicles are introduced.

**Fig. 5:**
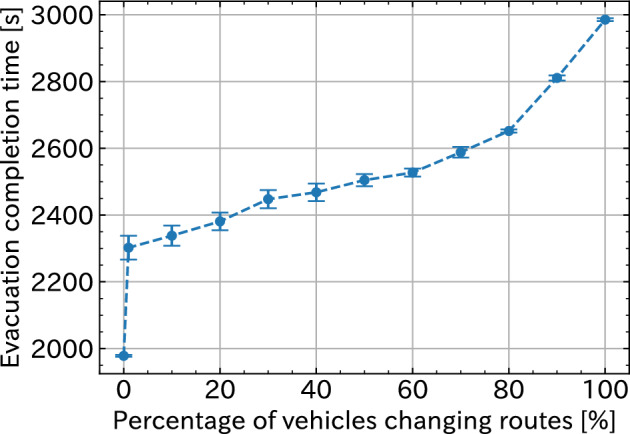
Change in evacuation completion time under a realistic scenario. The plot shows the evacuation completion time as a function of the fraction of vehicles that ignore the optimized routes and instead choose the shortest route to the nearest shelter. Markers represent averages over multiple simulation instances, and the associated error bars indicate the standard error. Even when only 1% of vehicles deviate from the optimized routes, evacuation efficiency degrades sharply, and thereafter the evacuation completion time increases almost monotonically as the deviation rate increases.

Specifically, starting from the solution obtained in Fig. 3b, we modify the routes and rerun the SUMO simulation to measure the evacuation completion time. Recall that four candidate routes are prepared for each vehicle, one of which is the route that follows the shortest path to the nearest shelter (Shortest). Among the vehicles that do not choose Shortest in the solution of Fig. 3b, we randomly select a subset and force those vehicles to take Shortest instead. The original solution prior to modification is taken to be the one obtained by the D-Wave Hybrid CQM solver, and we vary the number of vehicles whose routes are forcibly changed. The results are shown in Fig. 5. The horizontal axis indicates the fraction of vehicles that are switched to Shortest. The point at 0% corresponds to the idealized scenario in Fig. 2b, and the point at 100% corresponds to the All Shortest baseline. The results indicate that even when only 1% of vehicles deviate from the optimized routes, evacuation efficiency deteriorates sharply. This suggests that the solution obtained by the solver lies in a narrow and steep valley of the energy landscape, so that a small perturbation in the solution causes a substantial increase in energy. Developing methods to obtain more robust solutions that can mitigate such sensitivity remains an important topic for future work.

## Discussion

In this section, we first discuss a limitation of the proposed BQP/QUBO formulation itself, and then turn to the additional limitations introduced by the decomposition method. An important limitation of the formulation is the mismatch between the optimization objective and the final evaluation metric. The proposed objective minimizes distance-to-safety and route-overlap penalties, whereas evacuation completion time is measured only afterward through traffic simulation. Therefore, even when the original non-decomposed problem is solved, the obtained solution is not guaranteed to be optimal with respect to evacuation completion time itself. At the same time, the non-decomposed results indicate that this surrogate formulation can still reduce evacuation completion time relative to the baseline in the tested scenarios. We next discuss the factors that further limit performance when the decomposition method is introduced. A primary cause lies in the clustering strategy used to decompose the original QUBO. In this study, vehicles were grouped into clusters solely based on their two-dimensional spatial coordinates. While geographically close vehicles are likely to share candidate routes, this criterion does not explicitly account for the strength of interactions defined in the QUBO, namely the overlap of road segments between routes of different vehicles. As a result, vehicles that strongly interact through shared road segments can be assigned to different clusters. When this occurs, the interaction costs between such vehicles are not captured within the sub-QUBOs but instead appear as cross-cluster interaction terms during the merging step. Consequently, even if each sub-QUBO is solved well in isolation, the combined solution can incur a large penalty due to interactions across clusters. This explains why the merged solution exhibits a higher total cost than the non-decomposed solution, despite having low within-cluster costs.

**Fig. 6:**
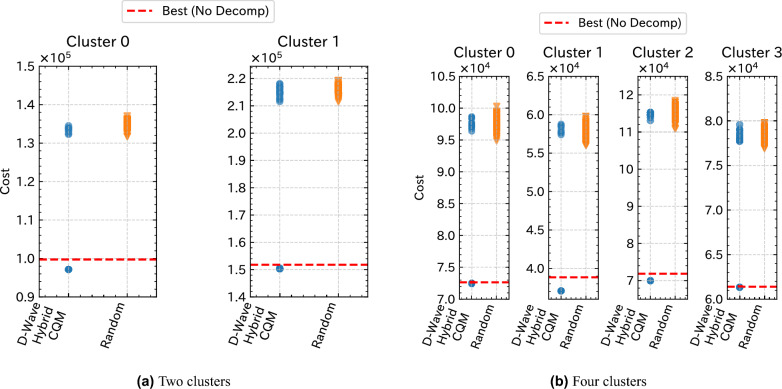
Cost comparison of sampled solutions for different numbers of clusters. (**a**) Two clusters and (**b**) four clusters. The red line (Best (No Decomp)) shows the reference cost obtained by partitioning the solution from the non-decomposed optimization into clusters and re-evaluating it using the cost function of the decomposition method. For each cluster, the decomposed CQM solver can generate solutions with lower cost than this reference. However, when these solutions are merged, the resulting global solution has a higher overall cost. This suggests that it is necessary to sample a diverse set of near-optimal solutions in each cluster.

In addition to the clustering method, the way candidate solutions are sampled and selected within each cluster also contributes to the degradation in performance. Figure 6 provides a detailed analysis of the costs of solutions sampled for individual clusters under different numbers of clusters. In this figure, the red line (Best (No Decomp)) represents a reference cost obtained by partitioning the solution from the non-decomposed optimization into clusters and re-evaluating it using the cost function of the decomposition method. As shown in Fig. 6, for each cluster, the decomposed CQM solver is able to generate solutions whose individual costs are lower than this reference value. However, when these individually low-cost solutions are combined across clusters, the resulting global solution exhibits a higher overall cost than the non-decomposed solution. This behavior indicates that the CQM solver tends to sample solutions with very low costs as well as relatively high-cost solutions for each sub-QUBO, while solutions with intermediate costs are sampled less frequently. Such intermediate-cost solutions may slightly increase the within-cluster cost but significantly reduce route overlaps with solutions selected in other clusters. Since these solutions are rarely included in the candidate set, the merging process is forced to combine solutions that are individually optimal within clusters but poorly compatible across clusters. As a result, the total cost of the obtained global solution becomes higher than that of the non-decomposed solution, which in turn leads to a degradation in evacuation completion time, as observed in Fig. 3b.

Furthermore, in this study, only the ten lowest-cost solutions were retained for each cluster. This fixed and small number limits the variety of route combinations available during the merging step. Increasing the number of retained solutions, or selecting solutions based not only on their individual costs but also on their route characteristics, such as overlap patterns with other clusters, may allow the merged solution to better balance within-cluster efficiency and cross-cluster congestion. A systematic examination of how the number and selection criteria of candidate solutions affect the final evacuation performance remains an important topic for future work.

In addition to these methodological issues, the present numerical experiments also involve several fixed modeling choices. In the numerical experiments, each vehicle was given four candidate routes, constructed as two candidate shelters and, for each shelter, a shortest route and a detour route. This setting was adopted as a minimum configuration that can represent both destination choice and route dispersion while keeping the candidate set size under control. However, the number of candidate routes is itself an important modeling parameter. If more candidate shelters or more route alternatives are introduced, the solution space becomes larger and different trade-offs between travel-distance reduction and congestion mitigation may emerge. Therefore, the sensitivity of the proposed method to the number and construction of candidate routes should also be investigated in future work.

A further practical issue for real-world deployment is that the recommended routes may not always coincide with the short-term objective of each individual driver. Therefore, the real-world effectiveness of the proposed method depends on the extent to which drivers follow the recommended routes. One possible direction is to improve public understanding of the benefit of system-wide optimization, namely the reduction in the overall evacuation completion time. In addition, if the method is implemented in an application, compliance rates could be recorded and linked to incentive mechanisms that encourage users to follow the recommended routes. Designing such compliance mechanisms is beyond the scope of the present study, but it will be important for practical deployment of evacuation guidance based on system-wide optimization.

In this work, we addressed a practical problem of evacuation route optimization in large-scale disasters by formulating it as a BQP and a QUBO, and by evaluating its performance using multiple solvers, including quantum annealers. The proposed method is designed to achieve both congestion avoidance and rapid evacuation by simultaneously minimizing the distance to safe areas and the route-overlap penalty among vehicles. Traffic simulations performed with SUMO showed that, when the BQP is solved without decomposition, the evacuation completion time can be reduced by up to 33.6% in the large-area model. This represents a substantial improvement over the locally optimal behavior in which all vehicles head to the nearest shelter along the shortest path, and provides strong evidence for the practical effectiveness of the proposed BQP formulation. On the other hand, the current D-Wave machines impose limits on the size of problems that can be embedded directly. To address this limitation, we proposed a decomposition method that leverages the sampling diversity of quantum annealing. The essential idea of the method is to transform the original large-scale BQP into an auxiliary quadratic selection problem over sampled candidate solutions of clustered sub-QUBOs. While the decomposition method offers the advantage of reducing computation time drastically, our results show that, when inter-cluster interactions are not sufficiently suppressed, the quality of the final solution falls short of the global optimum obtained without decomposition. This indicates that there is ample room for improvement in the decomposition strategy. In particular, incorporating clustering methods that explicitly account for the structure of interactions is likely to be crucial for improving performance. Furthermore, in a realistic scenario where some vehicles do not follow the optimized routes, we observed that the evacuation completion time deteriorates drastically even when only 1% of vehicles deviate from the optimized routes. This indicates that the obtained solution lies in a sharp valley of the energy landscape, reflecting the high sensitivity of large-scale evacuation routing problems. From a practical perspective, this observation suggests the importance of considering solution sensitivity and robustness in real-world deployment, rather than relying solely on strict optimality. Nevertheless, even under such deviations, the proposed method still achieves shorter evacuation completion times than the locally optimal baseline, which is an important and encouraging finding. Overall, the results of this study demonstrate that methods exploiting quantum annealing and its sampling diversity are promising tools for disaster response, where the primary requirement is to obtain high-quality solutions within strict time limits rather than to compute exact optima. An important direction for future research is to develop frameworks that explicitly account for robustness, such as intentionally sacrificing a small amount of optimality to favor solutions located in flatter regions of the objective landscape. Further research directions also include improving the decomposition method and bridging the gap between algorithmic design and real-world implementation. Moreover, because the proposed decomposition-based framework is not specific to evacuation optimization, it has the potential to be applied to a broader class of large-scale combinatorial optimization problems.

## Data Availability

The data used in this study were obtained from the Portal Site of Official Statistics of Japan (e-Stat) and OpenStreetMap. Hazard-related data and household distribution data were retrieved from e-Stat and processed by the authors. The code and other materials used to generate the simulation inputs are available from the corresponding author upon reasonable request.

## References

[1] Bayram, V. Optimization models for large scale network evacuation planning and management: A literature review. *Surv. Oper. Res. Manag. Sci.* **21**, 63–84. 10.1016/j.sorms.2016.11.001 (2016).

[2] The Asahi Shimbun. Fukushima daiichi genpatsu jiko, tōsho no keii [initial timeline of the fukushima daiichi nuclear accident]. Asahi Shimbun Digital (2011). (In Japanese). URL: https://www.asahi.com/special/10005/TKY201104010283.html [Accessed: 2025-08-19].

[3] Kadowaki, T. & Nishimori, H. Quantum annealing in the transverse Ising model. *Phys. Rev. E* **58**, 5355–5363. 10.1103/PhysRevE.58.5355 (1998).

[4] King, J. et al. Quantum annealing amid local ruggedness and global frustration. arXiv preprint arXiv:1701.04579 (2017). 1701.04579.

[5] Neukart, F. et al. Traffic flow optimization using a quantum annealer. *Front. ICT* **4**, 29 (2017).

[6] Inoue, D., Okada, A., Matsumori, T., Aihara, K. & Yoshida, H. Traffic signal optimization on a square lattice with quantum annealing. *Sci. Rep.* **11**, 1–12 (2021). 33568714 10.1038/s41598-021-82740-0 PMC7875976

[7] Ohzeki, M. *Reconsideration of optimization for reduction of traffic congestion*. *J. Phys. Soc. Jpn.* **2406**, 05448 (2024).

[8] Shikanai, R., Ohzeki, M. & Tanaka, K. Quadratic unconstrained binary formulation for traffic signal optimization on real-world maps. *J. Phys. Soc. Jpn.* **94**, 024001. 10.7566/JPSJ.94.024001 (2025).

[9] Hirama, S. & Ohzeki, M. Efficient algorithm for binary quadratic problem by column generation and quantum annealing. *J. Phys. Soc. Jpn.* **92**, 113002. 10.7566/JPSJ.92.113002 (2023).

[10] Takabayashi, T. & Ohzeki, M. Hybrid algorithm of linear programming relaxation and quantum annealing. *J. Phys. Soc. Jpn.* **93**, 034001. 10.7566/JPSJ.93.034001 (2024).

[11] Booth, M., Reinhardt, S. P. & Roy, A. Partitioning optimization problems for hybrid classical/quantum execution. Tech. Rep., D-Wave Systems Inc. (2017). URL: https://www.dwavequantum.com/media/jhlpvult/partitioning_qubos_for_quantum_acceleration-2.pdf.

[12] Zucca, A., Sadeghi, H., Mohseni, M. & Amin, M. H. *Diversity metric for evaluation of quantum annealing* **2110**, 10196 (2021).

[13] Karypis, G. & Kumar, V. *METIS: A Software Package for Partitioning Unstructured Graphs, Partitioning Meshes, and Computing Fill-Reducing Orderings of Sparse Matrices* (1998). University of Minnesota Technical Report.

[14] Lopez, P. A. et al. Microscopic traffic simulation using sumo. In *The 21st IEEE International Conference on Intelligent Transportation Systems* (IEEE, 2018).

[15] Gurobi Optimization, LLC. Gurobi Optimizer Reference Manual (2023).

[16] Jij-Inc. Openjij: Framework for the ising model and qubo (2025).

